# Use of Cognitive and Behavioral Strategies During a Weight Loss Program: A Secondary Analysis of the Doctor Referral of Overweight People to Low-Energy Total Diet Replacement Treatment (DROPLET) Trial

**DOI:** 10.1016/j.jand.2023.03.016

**Published:** 2023-10

**Authors:** John Aaron Henry, Nerys M. Astbury, Jamie Hartmann-Boyce, Constantinos Koshiaris, Susan A. Jebb

**Keywords:** Weight loss, Behavior change

## Abstract

**Background:**

Achieving a sustained energy deficit is essential for weight loss, but the cognitive and behavioral strategies that support this goal are unclear.

**Objective:**

The goal of this study was to investigate the number and type of cognitive and behavioral strategies used by participants who were enrolled in a 1-year weight loss trial and to explore associations between strategies and magnitude of weight loss at 3 months and 1 year.

**Design:**

The study is a secondary post-hoc exploratory analysis of data collected as part of the Doctor Referral of Overweight People to Low-Energy total diet replacement Treatment (DROPLET), a randomized controlled trial conducted in general practices in England, United Kingdom, between January 2016 and August 2017.

**Participants/setting:**

This study involved 164 participants from both intervention and control groups of the DROPLET trial who completed the Oxford Food and Behaviours (OxFAB) questionnaire to assess the use of 115 strategies grouped into 21 domains used to manage their weight.

**Interventions:**

Participants were randomized to either a behavioral weight loss program involving 8 weeks total diet replacement (TDR) and 4 weeks of food reintroduction or a program delivered by a medical practice nurse over a 3-month period (usual care [UC]).

**Main outcome measures:**

Weight was objectively measured at baseline, 3 months, and 1 year. Cognitive and behavioral strategies used to support weight loss were assessed using the OxFAB questionnaire at 3 months.

**Statistical analysis performed:**

Exploratory factor analysis was used to generate data-driven patterns of strategy use, and a linear mixed-effects model was used to examine associations between use of these patterns and weight change.

**Results:**

No evidence was found of a difference in the number of strategies (mean difference, 2.41; 95% confidence interval [CI], −0.83, 5.65) or the number of domains used (mean difference, −0.23; 95% CI, −0.69, 0.23) between the TDR group and the UC group. The number of strategies was not associated with weight loss at either 3 months (−0.02 kg; 95% CI, −0.11, 0.06) or 1 year (−0.05 kg; 95% CI, −0.14, 0.02). Similarly, the number of domains used was not associated with weight loss at 3 months (−0.02 kg; 95% CI, −0.53, 0.49) or 1 year (−0.07 kg; 95% CI, −0.60, 0.46).

Factor analysis identified four coherent patterns of strategy use, identified as Physical Activity, Motivation, Planned Eating, and Food Purchasing patterns. Greater use of strategies in the Food Purchasing (−2.6 kg; 95% CI, −4.42, −0.71) and Planned Eating patterns (−3.20 kg; 95% CI, −4.94, −1.46) was associated with greater weight loss at 1 year.

**Conclusions:**

The number of cognitive and behavioral strategies or domains used does not appear to influence weight loss, but the types of strategy appear of greater importance. Supporting people to adopt strategies linked to planned eating and food purchasing may aid long-term weight loss.

**Research Snapshot**

**Research Question:** What cognitive and behavioral strategies used by people for purposes of achieving weight loss are associated with success?

**Key Findings:** In a secondary analysis of a weight loss trial, the number of strategies used by participants was not associated with weight loss. However, there were associations between the patterns of strategy use and weight loss. Greater use of strategies in Food Purchasing and Planned Eating patterns was associated with greater weight loss at 1 year.

## Background

Excess adiposity is one of the major risk factors for preventable morbidity and mortality,^1^ and weight loss improves long-term health outcomes.^2^^,^^3^ Surveys suggest that approximately half of the population in England are attempting to lose weight at any one time.^4^

Behavioral weight management programs (BWMP) lead to greater weight loss than unsupported attempts.^5^ These programs typically combine multiple behavior change strategies to reduce energy intake or increase energy expenditure.^6^^,^^7^ However, there is considerable heterogeneity in the weight response both within and between participants in trials of BWMP.^7^ Whether such variations in response can be explained by use of specific behavioral strategies is unclear.

A recent review of long-term weight trajectories after weight loss programs found faster weight regain was associated with greater initial weight loss.^8^ However, few of the included papers in this review included sufficient detail on the intervention to characterize the content of the programs more precisely in terms of behavioral strategies used.

The Oxford Food and Activity Behaviours (OxFAB) taxonomy and questionnaire is a conceptual framework of 117 strategies grouped into 21 domains designed to provide details of the cognitive and behavioral strategies used during a weight loss attempt.^9^ Other taxonomies focus on the strategies that constitute a specific intervention from the perspective of the practitioner offering support,^10^^,^^11^ but OxFAB focuses on the cognitive and behavioral strategies employed by the individual.

The OxFAB taxonomy was constructed through a qualitative analysis of existing resources and a review of existing behavior change taxonomies and theories. The taxonomy was translated into a questionnaire to identify strategies used by individuals, and the validity of the questionnaire and test–retest reliability have been established previously.^9^ A cohort study following individuals undertaking a self-directed weight-loss attempt reported an association between weight loss at 3 months and the self-reported use of strategies relating to dietary impulse control, weight loss planning and monitoring, motivational support, information seeking, and self-monitoring.^12^

The aim of this study was to investigate the number and types of strategies used by participants enrolled in a weight loss trial and to analyze the associations between strategy use, participant characteristics, and weight change.

## Methods

### Study Design and Population

The Doctor Referral of Overweight People to Low-Energy total diet replacement Treatment (DROPLET) trial was a pragmatic, two-arm, parallel-group, open-label, individually randomized control trial. Eligible adults with a body mass index (BMI) of at least 30 and whose health would benefit from weight loss were invited by their general practitioner (GP) to participate. Those who had received or were scheduled for bariatric surgery, those participating in a weight management program, or those with contraindications to the total diet replacement (TDR) were excluded from participating (as detailed fully in the protocol).^13^ Two hundred seventy-eight adult participants with obesity who were seeking to lose weight were enrolled from primary care practices in Oxfordshire, England, United Kingdom, between January and July 2016, and follow-up was completed in August 2017. An independent statistician produced a computer-generated randomization list with 1:1 allocation to the TDR program or usual care (UC) groups using stratified block randomization with randomly permuted block sizes of 2, 4, and 6, stratified by general practice and BMI (≤35 or >35). After eligibility had been confirmed, participants were enrolled in the study, and the allocation was revealed using an online randomization program to ensure full allocation concealment.

### Low-Energy Total Diet Replacement Treatment

Participants met in-person with a counselor who had undergone training to deliver the behavioral weight loss support program involving TDR. For the first 12 weeks, participants met with the counselor in-person weekly for support, which comprised goal setting, feedback, encouragement, reassurance, and problem solving. Participants initially replaced all food with four portions of specially formulated food products daily (eg, soups, shakes, and bars), 750 mL of skim milk, 2.25 L of water or other low- or no-energy drinks, and a fiber supplement; energy intake was 810 kcal/day (3,389 kJ/day). Specially formulated foods were provided in discrete portions to participants by their counselor free of charge as part of the trial. After 8 weeks, there was a 4-week stepwise reduction in use of the specially formulated food products and reintroduction of conventional food-based meals. The counselors provided advice to participants on the kind of low-energy foods that should be consumed to maintain weight loss. During the weight maintenance phase from weeks 13 to 24, counselors encouraged participants to continue to meet with them monthly and to consume one specially formulated food product per day, with the remainder of the diet provided by conventional food. If weight was regained, the protocol allowed for participants to return to the TDR stage for up to 4 weeks. This program was free of charge to week 24.

### Usual Care Treatment

The UC program consisted of a series of appointments with a medical practice nurse over a 3-month period. Although the frequency and content of these sessions varied, all participating practices were provided with the same information and support to deliver a weight loss program, including advice and support to encourage a healthy energy-restricted eating plan using a booklet provided by the British Heart Foundation.^14^ This booklet contains tips and advice on ways to reduce energy intake and increase energy expenditure.

### Ethics Approval and Consent to Participate

The DROPLET trial protocol^13^ was reviewed and approved by South Central Oxford B NHS Research Ethics Service Committee (Ref 15/SC/0337). All participants who took part gave informed written consent before they were enrolled in the trial. The trial was prospectively registered with the ISRCTN registry (ISRCTN75092026).

### Sample Size

This is a secondary analysis of data collected as part of the DROPLET trial.^15^ The sample size for this study was not prospectively determined using a power calculation but was driven by the number of participants in the main trial who completed the OxFAB questionnaire and returned for follow-up measurements at 1 year (n = 164).

### Baseline Measures

All baseline measures were collected at participants’ medical practice during the baseline visit. Participants self-reported their age, sex (using two-response male/female options), and ethnicity (using the category options and free text fields as recommended by the UK government^16^) during an in-person baseline interview.

Home postcode (akin to zip code) was collected and used to determine participants’ Index of Multiple Deprivation (IMD), which was used as a marker of deprivation.^17^ The IMD ranks geographical areas of approximately 500 households in the United Kingdom on seven indices—income, employment, health deprivation and disability, education, crime, barriers to housing and services, and living environment. These ranks are grouped into deciles that were used for analysis, with lowest decile (1) representing the most deprived areas and highest decile (10) representing the least deprived areas.

Height was measured to the nearest 1 cm using stadiometers available in the practice, and weight was recorded to the nearest 0.1 kg using an electronic scale (SC-240 MA, Tanita Japan). Participants were asked to remove shoes and socks, wearing only light clothing for these measures.

### Follow-up Measures

All participants were invited to attend follow-up visits at their own general practice, where weight was measured at 3 months, 6 months, and 1 year after randomization.^15^

At the 3-month follow-up visit, participants completed the OxFAB questionnaire to assess the cognitive and behavioral strategies they used to manage their weight.^9^ The questionnaire consists of 117 questions, each assessing an individual strategy, grouped into 21 domains. The questionnaire was designed to have a binary outcome (use of a strategy or not), but to improve discrimination four response options were provided in the questionnaire. For each question, participants were asked to indicate whether they used the strategy “most of the time,” “sometimes,” “never/hardly ever,” or “not relevant to me.” Participants could also mark questions as “unclear.” Two questions were omitted from the questionnaire booklet used in the DROPLET trial because they were deemed not relevant in the context of a trial in which doctors referred individual participants (“I am losing weight with a friend/family member/my partner and I'm trying hard to lose more than them” and “I feel like I am part of a team with my friend(s)/partner/family member. We are losing weight together”). Therefore, for this study, a maximum of 115 strategies were grouped within 21 domains.

### Data Analysis

The analysis explored whether the use of strategies and domains influenced weight loss in the short (3 months) and longer term (1 year)***,*** evaluated whether the randomized treatment group and other characteristics (sex, age, weight at baseline, and IMD decile) influenced the cognitive and behavioral strategies adopted by participants, and tested whether data-driven and *a priori* patterns of strategy use were associated with weight loss in the short and long term.

Demographic characteristics and weight loss outcomes of those who completed the OxFAB questionnaire and other participants from the DROPLET trial who did not complete the questionnaire were compared using unpaired *t* test for continuous variables and *z* test of proportions for categorical variables.

Domain use was defined as the use of at least one strategy in which responses of “most of the time” and “sometimes” were deemed to indicate use of a strategy by a participant, and responses of “never/hardly ever” and “not relevant to me” considered evidence that participants did not use the strategy within a given domain.

The analyses used a multivariable linear regression model with treatment group, sex, age, baseline BMI, and IMD group (median split, most deprived IMD decile ≤8, least deprived IMD decile >8) as independent variables and total number of reported strategies, or domains as the dependant variable to explore associations between treatment group and participant characteristics on the number of OxFAB strategies and domains. The association between using specific strategies and domains was assessed using multivariable logistic regression, with strategy or domain use as the dependent variable, and treatment group, sex, age, baseline BMI, and IMD as independent variables.

A multivariable linear mixed-effects model including adjustment for fixed effects of treatment group, visit, strategy or domain use, age, sex, and IMD as well as interaction terms for strategy or domain use by visit and strategy or domain use by group and participant as a random effect explored the effect of strategy and domain use on weight change from baseline.

Exploratory factor analysis was used to identify patterns of strategy use as described in detail previously.^12^ Data-driven patterns of strategy use were derived by exploratory factor analysis for the binary strategy use variables, using a tetrachoric correlation matrix with orthogonal rotation (varimax option) to enhance factor identification and interpretability. Factors were retained based on eigenvalues >1 and the proportion of variance explained. For each factor identified, every strategy has a factor loading, which is the correlation coefficient between each strategy and the factor. Only the strategies with factor loadings (*z*-score) > 0.2 were retained and extracted in the factors identified.

The effects of using each of the identified strategy patterns on weight change from baseline was assessed using a multivariable linear mixed-effects model. This model included adjustment for fixed effects of the pattern use, visit, age, sex, IMD group, and baseline BMI, and interaction terms for pattern use by visit and pattern use by treatment group and a random effect of participant.

Finally, the study assessed whether the use of an *a priori* model of “essential weight loss strategies” was associated with weight loss. This model was developed previously and was shown to be associated with weight loss in previous work.^12^ The essential strategies model contained nine components made up of 31 strategies: food intake targets (one strategy); weight targets (two strategies); impulse management (seven strategies); motivation to lose weight (15 strategies); advance meal planning (one strategy); monitoring of food intake (two strategies); swapping less healthy foods for healthier ones (one strategy); keeping unhealthy food out of the house (one strategy), and self-weighing (one strategy). Each participant was judged to have used each component if they reported using one or more of the strategies contained within the component “most of the time.” A total score (0–9) was calculated, with each component receiving equal weighting. This score represented the number of components within the “essential strategies” model that each participant used. Weight loss in participants who reported using the “essential strategies” (defined as seven or more of nine of the components in this “essential” model) was compared with weight loss in those who did not report using the full group of “essential strategies” (less than seven out of nine), using multivariable linear regression adjusted with fixed factors to include essential strategy use, visit, treatment group, sex, age, baseline BMI and IMD decile, interaction terms of essential strategy use × visit and essential strategy use × treatment group and participant ID as random factor.

All data analysis was conducted in Stata 14SE.^18^ A *P* value of less than .05 was deemed to be statistically significant.

## Results

### Study Population

**Figure 1:**
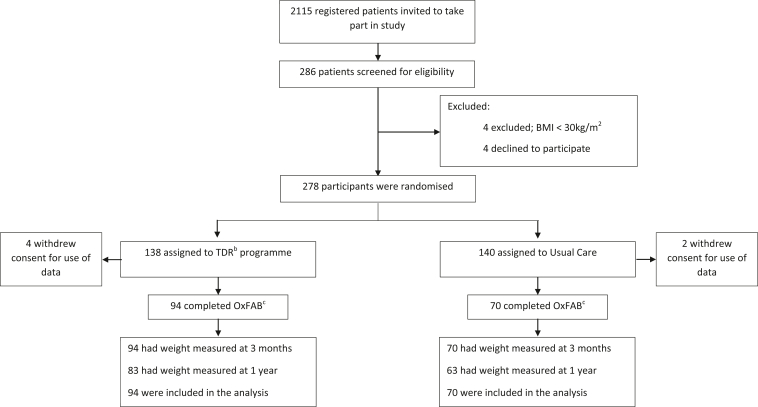
Flowchart of participants in the DROPLET^a^ trial. ^a^DROPLET = Doctor Referral of Overweight People to Low-Energy total diet replacement Treatment (DROPLET). ^b^TDR = Total Diet Replacement. ^c^OxFAB = Oxford Food and Behaviors questionnaire (OxFAB) assesses 117 (only 115 used in this study) cognitive and behavioral strategies, grouped into 21 domains used by individuals losing weight.

General practitioner practices sent letters to 2,115 registered patients, inviting them to take part. Two hundred seventy-eight participants were enrolled from 10 GP practices across Oxfordshire and randomly allocated to either a TDR program or usual care. Six participants (four from the intervention group and two from the control group) withdrew their consent for use of their data after they had enrolled. Of the 272 participants enrolled in the main trial for whom we can use data, 164 (59%) participants completed the OxFAB questionnaire at 3 months, with 94 of these participants in the TDR arm and 70 in the UC arm (57% and 43% of all those enrolled in the TDR and UC groups, respectively) (Fig 1). The participants who completed the OxFAB questionnaire were slightly older and had lower baseline BMI, and there was a greater proportion from the TDR treatment group and from (self-reported) white ethnicity categories compared with the participants who did not complete the OxFAB questionnaire (Table 1)*.*

**Table 1:** Demographic characteristics of participants enrolled in the DROPLET^a^ trial at baseline

Characteristic	All DROPLET participants,^b^ n = 272	Did not complete OxFAB,^c^ n = 108	Completed the OxFAB, n = 164	Between-group differences^d^
Treatment group, n (%)				.001
TDR^e^	134 (49%)	40 (37%)	94 (57%)	
Usual care	138 (51%)	68 (63%)	70 (43%)	
Age, y; mean (SD)	47.8 (12.1)	44.3 (12.8)	50.2 (11.2)	<.001
Sex, n (%)				.701
Female	165 (61)	64 (59)	101 (62)	
Male	107 (39)	44 (41)	63 (38)	
Ethnicity, n (%)^f^				*P* < .01
White	250 (91.9)	91 (84.3)	159 (97.0)	*P* < .01
Mixed or multiple ethnic groups	4 (1.47)	4 (3.7)	0	.01
Asian	12 (4.4)	8 (7.4)	4 (2.4)	.05
Black, Caribbean or African	4 (1.5)	3 (2.8)	1 (0.6)	.15
Other^g^	2 (0.8)	2 (2)	0 (0)	.08
IMD decile^h^	7.4 (1.9)	7.0 (1.9)	7.7 (1.9)	<.01
Weight (kg), mean (SD)	106.5 (19.4)	108.3 (21.1)	105.0 (17.9)	.21
Height (cm), mean (SD)	169.0 (9.6)	168.4 (9.9)	169.3 (9.4)	.45
BMI, mean (SD)	37.2 (5.4)	38.12 (6.5)	36.6 (4.5)	.02
Weight change 3 months (kg), mean (SD)	−3.8 (3.8)	−2.3 (3.3)	−4.7 (3.8)	<.01
Weight change 12 months (kg),^i^ mean (SD)	−5.8 (8.3)	−2.9 (6.2)	−7.7 (9.1)	<.01

a Doctor referral of overweight people to low-energy total diet replacement Treatment (DROPLET).

b Data are presented for all participants for whom we had consent to use their data (n = 272). Although 278 participants were enrolled in the DROPLET trial (n = 138 TDR, n = 140 Usual Care), six participants withdrew from the study and also withdrew their consent for us to use their data.

c Oxford Food and Behaviours (OxFAB) questionnaire.

d Participants who completed the OxFAB questionnaire and those who did not were compared using unpaired *t* test for continuous variables and using *z* test of proportions for categorical variables.

e TDR = total diet replacement.

f Ethnicity was self-reported by participants in an in-person interview with the medical practice nurse at the baseline visit in the categories used in the UK census.

g All participants who did not report being in one of the above categories were grouped under “Other” ethnicity category heading.

h Index of Multiple Deprivation (IMD); The IMD ranks geographical areas of about 500 households in the UK on seven indices: income, employment, health deprivation and disability, education, crime, barriers to housing and services, and living environment. These ranks are grouped into deciles, which were used for analysis with lowest decile (1) representing the most deprived areas and highest decile (10) representing the least deprived areas.

i Weight change from baseline analyzed using last observation carried forward (LOCF) substitution for any missing follow-up data at 1 year.

### Weight Loss

Of the participants who completed the OxFAB questionnaire, mean weight change at 3 months was −6.5 kg (standard deviation [SD], 3.7) in the TDR group and −2.2 kg (SD, 2.3) in UC, and at 1 year was −10.9 kg (SD, 9.6) and −3.4 kg (SD, 6.1) in TDR and UC, respectively. The participants who completed the OxFAB questionnaire on average lost more weight at 3 months and 1 year than the participants who did not complete the OxFAB questionnaire (Table 1).

### Strategy and Domain Use

The number of participants reporting use of each individual strategy varied considerably. The mean number of strategies used by participants some or most of the time was 53 (SD, 19) out of a maximum 115. Two strategies were used by more than 80% of participants: “I have a goal weight in mind that I am working toward” and “To help me manage what I eat, I try to avoid certain foods.” In contrast, four strategies were used by fewer than 10% of participants: “I exercise with a group and when I don’t attend someone asks me why I wasn’t there”; “I have an online weight loss buddy”; “I put aside money at the beginning of my weight loss attempt and only allow myself to have it back if I’ve hit my targets”; and “I use something at my desk that helps me stay more active when working (eg, a standing desk).”

When defined as use of at least one strategy within that domain, the mean number of domains used by participants was 17 (SD, 3.1) out of a maximum of 21. “Goal setting,” “Regulation: Rule Setting,” and “Self-monitoring” were the most commonly used domains, with over 95% of participants reporting using at least one strategy within each of those domains. Two domains, “Imitation (modeling)” and “Support: Buddying,” were used by fewer than half of participants (Supplemental Figure 2).

### Strategy and Domain Use between Trial Arms

No evidence was found of a difference in the number of strategies used (mean difference, 2.41; 95% confidence interval [CI], −0.83, 5.65) or the number of domains used (mean difference, −0.23; 95% CI, −0.69–0.23) between those in the TDR compared with UC group (Table 2). However, there were differences in the types of domains used between groups. Participants in the TDR group had greater odds of reporting using strategies in the information seeking, reward, support: motivational, and use of weight management aids domains and lower odds of reporting using strategies in the Imitation (modeling), impulse management: awareness of motives, impulse management: distraction, regulation: allowances, support: buddying, and support: professional domains compared with the UC group (Table 3).

**Table 2:** Association between number of OxFAB^a^ strategies and domains used and baseline characteristics of participants in the DROPLET^b^ trial

Characteristics	Total Number of Strategies Used		Total Number of Domains Used	
	β	(95% CI^c^)	β	(95% CI^c^)
**Model 1**^d^				
Treatment group^e^	2.97	(0.04–5.90)	−0.18	(–0.66–0.29) 0.291764694
**Model 2**^f^				
Treatment group^e^	3.49	(0.68–6.30)	−0.10	(–0.56–0.36)
**Model 3**^g^				
Treatment group^e^	2.44	(−0.36–5.24)	−0.23	(−0.69–0.23)
**Model 4**^h^				
Treatment group^e^	2.35	(−0.45–5.15)	−0.23	(−0.69–0.23)
**Model 5**^i^				
Treatment group^e^	2.41	(−0.83–5.65)	−0.23	(−0.69–0.23)
Sex^j^	−10.47	(−13.74 to −7.19)	−1.66	(−2.13 to −1.2)
Age (years)	−0.28	(−0.43 to −0.14)	−0.04	(−0.06 to −0.02)
Baseline BMI	0.22	(−0.14–0.58)	0.00	(−0.05 to 0.05)
IMD decile^k^	−1.71	(−4.98–1.56)	−0.03	(−0.5 to 0.43)
Constant	62.05	(45.93–78.17)	19.12	(16.83–21.41)

a Oxford Food and Behaviours questionnaire (OxFAB) assesses 117 (only 115 used in this study) cognitive and behavioral strategies, grouped into 21 domains used by individuals losing weight.

b Doctor Referral of Overweight People to low-energy total diet replacement Treatment (DROPLET).

c CI = confidence interval.

d Model 1: Multivariable linear regression model with number of strategies/domains used as outcome variable, main exposure: treatment group, unadjusted.

e Randomized treatment group (Total diet replacement [TDR or Usual Care] [Reference group = Usual Care]).

f Model 2: Multivariable linear regression model with number of strategies/domains used as outcome variable, main exposure: treatment group, adjusted for covariates: sex.

g Model 3: Multivariable linear regression model with number of strategies/domains used as outcome variable, main exposure: treatment group, adjusted for covariates: sex and age.

h Model 4: Multivariable linear regression model with number of strategies/domains used as outcome variable, main exposure: treatment group, adjusted for covariates: sex, age, and baseline BMI.

i Model 5: Multivariable linear regression model with number of strategies/domains used as outcome variable, main exposure: treatment group, covariates: sex, age, baseline BMI, and IMD decile.

j Self-reported sex using binary (male/female) options (reference group = female).

k Index of Multiple Deprivation (IMD); The IMD ranks geographical areas of approximately 500 households in the United Kingdom on seven indices: income, employment, health deprivation and disability, education, crime, barriers to housing and services, and living environment. These ranks are grouped into deciles, which were used for analysis, with lowest decile (1) representing the most deprived areas and highest decile (10) representing the least deprived areas.

**Table 3:** Association between use of strategies within the domains of the OxFAB^a^ and demographic characteristics of participants in the DROPLET^b^ trial^c^

Domain	Treatment group	Sex	Age	Baseline BMI	IMD^d^
	OR (95% CI)	OR (95% CI)	OR (95 % CI)	OR (95% CI)	OR (95% CI)
Energy compensation	0.80 (0.53–1.19)	0.53 (0.36–0.79)	0.98 (0.96–1.00)	1.00 (0.95–1.04)	1.30 (0.86–1.96)
Goal setting	0.71 (0.33–1.55)	0.20 (0.09–0.46)	0.95 (0.92–0.99)	0.97 (0.89–1.05)	4.65 (1.56–13.83)
Imitation (modeling)	0.55 (0.39–0.77)	0.70 (0.50–0.99)	0.99 (0.97–1.00)	0.98 (0.95–1.02)	0.76 (0.54–1.07)
Impulse management: acceptance	0.94 (0.65–1.35)	0.97 (0.67–1.39)	1.00 (0.98–1.02)	1.00 (0.96–1.04)	0.97 (0.67–1.39)
Impulse management: awareness of motives	0.63 (0.44–0.90)	0.38 (0.27–0.54)	0.99 (0.97–1.01)	1.00 (0.97–1.04)	1.03 (0.72–1.47)
Impulse management: distraction	0.47 (0.27–0.82)	0.48 (0.28–0.80)	0.93 (0.90–0.95)	1.05 (0.98–1.12)	1.07 (0.63–1.83)
Information seeking	2.06 (1.04–4.08)	0.13 (0.06–0.29)	0.96 (0.93–0.99)	0.94 (0.88–1.0)	0.82 (0.41–1.64)
Motivation	0.52 (0.25–1.07)	0.26 (0.13–0.52)	0.97 (0.94–1.01)	1.02 (0.95–1.11)	0.89 (0.45–1.75)
Planning content	1.03 (0.55–1.95)	0.31 (0.16–0.60)	0.96 (0.93–0.99)	0.91 (0.86–0.98)	1.00 (0.51–1.93)
Regulation: allowances	0.34 (0.24–0.48)	0.60 (0.42–0.84)	1.01 (0.99–1.02)	1.05 (1.01–1.09)	1.28 (0.90–1.82)
Regulation: restrictions	0.65 (0.39–1.08)	1.14 (0.69–1.90)	0.96 (0.94–0.98)	1.02 (0.96–1.08)	0.90 (0.55–1.48)
Regulation: rule setting	0.99 (0.41–2.38)	0.65 (0.27–1.53)	0.94 (0.89–0.98)	0.84 (0.77–0.92)	e
Restraint	0.94 (0.67–1.34)	0.58 (0.41–0.82)	1.01 (0.99–1.02)	0.92 (0.89–0.96)	1.32 (0.93–1.88)
Reward	2.27 (1.60–3.24)	0.25 (0.18–0.36)	0.98 (0.96–0.99)	1.07 (1.03–1.12)	1.01 (0.71–1.45)
Scheduling	0.61 (0.36–1.04)	0.43 (0.26–0.70)	1.01 (0.98–1.03)	1.01 (0.95–1.07)	1.80 (1.04–3.12)
Self-monitoring	0.64 (0.18–2.21)	1.42 (0.41–4.86)	1.01 (0.96–1.06)	0.95 (0.84–1.08)	e
Stimulus control	0.61 (0.36–1.04)	0.43 (0.26–0.70)	1.01 (0.98–1.03)	1.01 (0.95–1.07)	1.80 (1.04–3.12)
Support: buddying	0.61 (0.43–0.88)	1.59 (1.11–2.28)	0.99 (0.98–1.01)	1.05 (1.01–1.09)	0.42 (0.29–0.62)
Support: motivational	2.49 (1.24–5.01)	0.11 (0.05–0.24)	1.02 (0.98–1.05)	0.92 (0.86–0.99)	0.29 (0.14–0.58)
Support: professional	0.48 (0.25–0.92)	0.17 (0.08–0.33)	0.96 (0.94–0.99)	1.12 (1.03–1.22)	0.48 (0.26–0.89)
Weight management aids	8.64 (5.71–13.07)	0.89 (0.60–1.34)	0.96 (0.94–0.98)	1.00 (0.96–1.04)	0.66 (0.44–0.99)

a Oxford Food and Behaviours (OxFAB) questionnaire.

b Doctor Referral of Overweight People to low-energy total diet replacement Treatment (DROPLET).

c Comparator groups were: Usual Care (treatment group), female (sex), IMD decile ≥ 8 (least deprived regions), age, and baseline BMI were used as continuous variables; thus, results indicate OR for a one-unit increase in these variables.

d Index of Multiple Deprivation (IMD) is calculated using participant postcode. Higher IMD deciles indicate least-deprived regions, and lower IMD deciles indicate more deprived location.

e Insufficient number of participants within cells to conduct analysis.

### Associations Between Total Number of Strategies and Domains Used and Participant Characteristics

Women reported using significantly more strategies (mean difference, −10.47; 95% CI, −13.74, −7.19) and domains (mean difference, −1.66; 95% CI, −2.13, −1.2) compared with men. Older people tended to use fewer strategies and domains, with each additional year of age associated with use of 0.3 fewer strategies (95% CI, −0.43, −0.14) and 0.04 fewer domains (95% CI, −0.06, −0.02) (Table 2). No evidence was found that the self-reported number of strategies or domains used was associated with participants’ baseline BMI or that there were differences in the number of strategies or domains used between those from the least and most deprived areas (Table 2).

### Association Between Domain Use and Participant Characteristics

Men had lower odds of reporting strategies in the energy compensation, goal setting, imitation (modeling), impulse management: awareness of motives, impulse management: distraction, information seeking, motivation, planning content, regulation: allowances, restraint, reward, scheduling, stimulus control, support: motivational, and support: professional domains, but greater odds of reporting strategies in the support: buddying domain than the women (Table 3).

Older participants had lower odds of reporting the use of strategies in the goal setting, impulse management: distraction, information seeking, reward, planning content, regulation rule setting, support: professional, and weight management aids domains compared with their younger counterparts (Table 3).

Participants with a higher baseline BMI had lower odds of reporting the use of strategies in the planning content, regulation: rule setting, and restraint domains compared with those with lower baseline BMI and greater odds of reporting the use of strategies in the regulation: allowances, reward, support: buddying and support: professional domains compared with those of lower baseline BMI (Table 3).

Participants from the most deprived regions had almost five times the odds of reporting the use of strategies from the goal setting domain and approximately two times the odds of reporting use of strategies in the scheduling and stimulus control domains (Table 3). However, participants from these most deprived regions had lower odds of reporting using strategies in the buddying, motivational support, professional support, and use of weight management aids domains than participants in the least deprived areas (Table 3).

### Associations Between Strategy and Domain Use and Weight Change

There was no evidence that the number of strategies used was associated with weight loss at 3 months (−0.02 kg; 95% CI, −0.11, 0.06), or 1 year (−0.05 kg; 95% CI, −0.14, 0.02). Similarly, no evidence was found that the number of domains used was associated with weight change at either time point (−0.02 kg; 95% CI, −0.53, 0.49 and −0.07 kg; 95% CI, −0.60, 0.46 at 3 months and 1 year, respectively). Self-reported use of some specific strategies was associated with weight loss at 3 months and 1 year (Supplementary Fig 3).

No associations were found with the use of specific domains and weight change at 3 months, but use of some specific domains was associated with weight change at 1 year. Participants who reported the use of goals, motivation, and use of weight management aids domains lost more weight at 1 year than their counterparts who did not report using strategies within these domains (Table 4).

**Table 4:** Associations between domain use reported using the OxFAB^a^ questionnaire and weight change from baseline in participants in the DROPLET^b^ trial

Domain	Weight Change From Baseline to 3 Months^c^		Weight Change From Baseline to 1 Year^c^	
	Mean difference (kg)	95% CI	Mean difference (kg)	95% CI
Energy compensation	0.51	(−3.39–4.41)	1.03	(−3.00–5.06)
Goal setting	−3.70	(−10.92–3.52)	−7.86	(−15.09 to −0.63)
Imitation (modeling)	−0.19	(−3.47–3.10)	2.01	(−1.37–5.39)
Impulse management: Acceptance	1.96	(−1.66–5.58)	−0.60	(−4.37–3.17)
Impulse management: Awareness of motives	1.43	(−1.96–4.82)	0.01	(−3.50–3.51)
Impulse management: Distraction	−0.27	(−5.26–4.71)	−4.33	(−9.50–0.85)
Information seeking	−0.94	(−7.45–5.56)	−3.83	(−10.82–3.15)
Motivation	−4.78	(−11.28–1.72)	−6.95	(−13.46 to −0.44)
Planning content	−0.70	(−6.94–5.54)	−1.85	(—8.77–5.07)
Scheduling of diet and activity	2.29	(−0.98–5.56)	3.65	(0.27–7.02)
Regulation: Allowances	4.02	(−0.84–8.88)	5.06	(0.02–10.10)
Regulation: Restrictions	−0.77	(−9.08–7.55)	1.91	(−6.91–10.73)
Regulation: Rule setting	−1.73	(−5.09–1.62)	−0.09	(−3.57–3.39)
Restraint	−0.88	(−4.11–2.34)	0.42	(−2.90–3.73)
Reward	−1.10	(−5.98–3.78)	−1.35	(−6.42–3.72)
Self-monitoring	−2.11	(−13.75–9.54)	−5.85	(−17.05–5.80)
Stimulus control	−1.10	(−5.98–3.78)	−1.35	(−6.42−3.72)
Support: Buddying	−0.15	(−3.65–3.35)	0.04	(−3.59–3.67)
Support: Motivational	−3.29	(−9.80–3.22)	−4.38	(−11.37–2.61)
Support: Professional	0.81	(−4.97–6.59)	2.20	(−3.60–8.00)
Weight management aids	−1.18	(−4.60–2.25)	−3.69	(−7.26 to −0.12)

a Oxford Food and Behaviours (OxFAB) questionnaire.

b Doctor Referral of Overweight People to low-energy total diet replacement Treatment (DROPLET).

c Reported as weight change from baseline (kg) in the participants reporting use of the domain compared with the participants who did not report use the domain.

### Identifying Patterns of Strategy Use

The exploratory factor analysis showed five distinct patterns of strategy use. Four of these patterns were conceptually coherent and were defined as Physical Activity, Motivation, Planned Eating, and Food Purchasing behaviors (Supplementary Fig 4).

One of the strategies within the pattern we named “Food Purchasing” did not fit exactly with food purchasing behavior (“I exercise with a group and when I don’t attend someone asks me why I wasn’t there”), but the factor loading score for this strategy was only just above the loading score threshold (0.2), and it was considerably lower than for the other strategies within the same pattern, so we disregarded this strategy when naming the pattern.

### Association Between Strategy Patterns and Weight Change

Evidence was seen of an association between use of the “Food Purchasing” and “Planned Eating” strategy patterns and weight loss. Greater use of strategies from the “Planned Eating” pattern was associated with an additional −3.20 kg (95% CI, −4.94, −1.46) weight loss at 1 year. Greater use of strategies in the “Food Purchasing” pattern was associated with an additional −2.6 kg (95% CI, −4.42, −0.71) weight loss at 1 year (data not shown).

### Association Between Use of the *A Priori* Strategy Model and Weight Change

The mean number of strategies from the *a priori* model used by participants was four out of a maximum of nine (SD, 2.5). No association was seen between the number of strategies in the model and weight loss at 3 months (mean weight loss, −0.35 kg; 95% CI, −0.96–0.24). However, within the model, each additional strategy used was associated with an additional −1.22 kg (95% CI, −1.85 to −0.58) weight loss at 1 year. Participants who used seven or more of the strategies in the “essential weight loss model” did not lose any more weight at 3 months (mean weight change, −1.20 kg; 95% CI, −5.09–2.68), although use of seven or more strategies from the essential model was associated with an additional −4.49 kg (95% CI, −8.55 to −0.45) weight loss at 1 year compared with those who used fewer than seven of the nine strategies in this model (data not shown).

## Discussion

This study used the OxFAB questionnaire to explore the behavioral and cognitive strategies used by participants who were undertaking a weight loss attempt as part of a randomized controlled trial. No association was found between the total number of strategies or domains used and weight loss, but strategies linked to food purchasing and planned eating were associated with successful weight loss. Adherence to an *a priori* model of essential weight loss strategies was also associated with greater weight loss.

None of the strategies was associated with weight loss at 3 months; however, participants who reported using strategies in the goal setting, motivation, and use of weight management aids domains lost more weight than participants who did not report using these strategies at 1 year. Possibly the strategies that confer greater weight loss in the short term may be different from those that best aid with weight loss maintenance in the longer term.^19^

The results here are broadly consistent with the findings of a short-term cohort study using the same questionnaire in individuals who were attempting to self-manage their body weight.^12^ The effective strategies varied, perhaps reflecting the difference between self-help approaches and the formal weight-loss programs studied here. Indeed, differences in the use of weight loss strategies in the two trial arms largely reflect the nature of the programs. Participants in the UC group reported varied use of strategies, whereas participants who were randomized to the TDR intervention had greater odds of reporting using strategies in the domains of information seeking, reward, support: motivational, and use of weight loss aids. However, of the domains more likely to be used by the TDR group, the use of strategies within these domains was not associated with weight loss at 3 months. Only the use of strategies in the weight loss aids domain was associated with significant weight loss at 1 year.

The strategies associated with weight loss at 1 year are consistent with observational data from the US National Weight Control Registry (NWCR), a self-selected population of more than 4,000 individuals who have lost at least 13.6 kg (30 lb) and kept it off for at least 1 year.^20^ Participants in the NWCR report that strategies relating to food (eating a low-calorie, low-fat diet, eating breakfast regularly, and maintaining a constant eating pattern) were important for weight maintenance after weight loss.^21^

The findings from the current study indicate a striking difference between participants from the most and least deprived regions, with the former having 50% lower odds of seeking external support to manage their weight. This may reflect differences in awareness of support available or financial or other resource constraints that could limit to access these support systems. A recent review of strategies associated with weight change after behavioral weight loss programs found that the opportunity to access programs, usually involving additional support, after the initial weight loss phase was associated with slower weight regain.^8^ This access to ongoing support may be contributing to disparities in health outcomes after weight loss interventions.

A strength of this study is that it used a questionnaire specifically developed and validated to assess the behavioral strategies used during weight loss. However, one of the limitations of the questionnaire is that it contains 117 questions (of which 115 were used in this study) and takes at least 15 minutes to complete. We believe that one consequence of its lengthy nature is that when the questionnaire was included in the package of questionnaires provided to participants in the DROPLET trial at 3 months, only 164 of the 278 (59%) participants completed it. This is similar to a cohort study that used the OxFAB questionnaire to assess cognitive and behavioral strategies used by people engaging in a self-directed weight loss attempt in which a similar proportion of participants failed to complete the questionnaire.^12^

The OxFAB questionnaire is continuously evolving, and to address issues on excessive length the authors recently reduced the existing 117-item questionnaire to 20 items.^22^

Limitations include that the analyses relied on converting ordinal variables into binary strategy use. However, the OxFAB questionnaire was designed to be analyzed using dichotomous outcomes (use of a strategy or not), but during the refinement process, participants provided feedback saying that it was difficult for them to provide a yes/no response, so additional response options were added, which made it easier for participants to respond while still allowing researchers to analyze the strategies and domains in a dichotomous manner.^9^ Further limitations include the nature of multiple tests, and the possibility that some findings occurred by chance. Nevertheless, the data reduction technique used for multiple exposures could be considered a strength to the approach.

One further limitation to this study is the generalizability of the findings. The participants in the DROPLET trial were mainly white (British) and middle-aged, and from areas with low levels of deprivation, which, although not representative of the UK population as a whole, is representative of the local population. The people who completed the questionnaire were older and had lower baseline BMI, and there were also a greater proportion from the TDR treatment group and who self-reported to be of white ethnicity than those who didn’t complete the questionnaire. These differences may go some way to explain why differences in weight loss were observed between those who completed and did not complete the questionnaire. The provision of binary option for gender may have discouraged participation from nonconforming gender individuals or may have caused them to misclassify their gender. Nevertheless, the participants who completed the OxFAB questionnaire on average lost more weight at both 3 months and 1 year compared with the participants who did not complete the OxFAB questionnaire.

Although there were differences in age, baseline BMI, treatment group, and ethnicity between the people who completed the OxFAB questionnaire and those who did not, which may explain differences in weight loss between the groups, it is also possible, and perhaps more likely, that the people who were willing to take the time to complete the questionnaire represent a more motivated group of people than those who did not. Differences in motivation could explain why these people were more successful in their weight loss attempt. However, we cannot exclude the possibility that the results of this sample reflect the strategies used by people that are more successful than the population as a whole, or the possibility that those who did not complete the questionnaire used different strategies than those who provided data in this study. The findings reported here cannot be assumed to be causal, but they may provide useful insights for the development of future programs. It is difficult to rigorously assess the internal validity of questionnaires such as this. In particular, self-report may not be reliable; however, the face/validity and repeatability of the questionnaire has been previously established,^9^ although we cannot rule out some reverse causality with recollections of strategy use influenced by the outcome of the weight loss attempt.

## Conclusions

The number of cognitive and behavioral strategies or domains does not appear to influence weight loss; types of strategy appear of greater importance. Supporting people to adopt strategies linked to planned eating and food purchasing may aid long-term weight loss.

## Figures and Tables

**Supplementary Figure 2:** Frequency of strategies within the domains of the OxFAB^a^ questionnaire used by participants from the DROPLET^b^ trial.

Domain	Definition of strategies	Proportion of participants reporting use of the strategy^c^	Proportion of participants reporting use of at least one strategy in the domain^d^	Mean proportion of strategies used within the domain^e^
		N (%)	%	Mean (SD)
Energy compensation	I try to balance how much I eat, so if I eat a lot at one point I’ll make up for it by eating less at other points	121 (74)	80	40 (31)
	I adjust how much I eat depending on how much physical activity I’ve done or plan to do	71 (43)		
	I adjust how much physical activity I do depending on how much I’ve eaten or am planning to eat later	31 (19)		
	If I haven’t been very active, or know I’m going to be inactive for a while, I’ll exercise more at another point to balance it out	42 (26)		
Goal setting	I set myself goals for what or how much I’ll eat each day or each week	94 (57)	95	61 (26)
	I set myself goals for how active I’ll be each day or each week	65 (40)		
	I know how I want to look and won’t give up losing weight until I am the size and shape I want to be	125 (76)		
	I have a goal weight in mind that I am working toward	142 (87)		
	I set goals that may be unrealistic	84 (51)		
	I want to achieve my weight loss targets within a set amount of time or by a certain date (eg, I want to lose weight in time for my holiday)	99 (60)		
	I have a clear goal for the amount of weight I want to lose each week	94 (57)		
Imitation (modeling)	I’ve decided to eat some particular foods or do certain types of physical activity in my attempt to lose weight because it worked for someone I know	58 (35)	35	35 (48)
Impulse management: Acceptance	When I feel like eating something that doesn’t fit with my diet, I acknowledge the feeling but don’t act on it	110 (67)	75	48 (34)
	When I feel uncomfortable or am in pain when I’m exercising, I accept it as I know it’s doing me good	49 (30)		
Impulse management: Awareness of motives	If I feel like eating but am trying not to, I pause and ask myself if I’m hungry	89 (54)	69	40 (34)
	Partway through eating a meal, I pause and ask myself if I’m full enough to stop	70 (43)		
	If I feel like being inactive, I ask myself why I feel that way	40 (24)		
Impulse management: Distraction	If I feel like eating but am trying not to, I find something else to do to distract myself	126 (77)	89	56 (29)
	When I have a craving for something I shouldn’t eat, I try to think about something else to take my mind off it	123 (75)		
	If I feel like eating but am trying not to, I make myself wait a certain amount of time to see if the craving passes	103 (63)		
	If I feel like eating but am trying not to, I chew gum, drink something, or brush my teeth to stop me from doing so	96 (59)		
	I try to go to sleep as a way to not eat when I am feeling hungry	49 (30)		
	If I feel like I want to stop exercising, I focus on something else to distract me so that I keep going	55 (34)		
Information seeking	When I’m grocery shopping and items of food look similar, I make my choice based on the nutritional information on the food labels	115 (70)	92	52 (28)
	I use a book, website, or app to look up the nutrition information or calorie content of the foods I eat	81 (49)		
	I’ve looked up information on how many calories I burn doing physical activity	50 (30)		
	I’ve looked up strategies, tips, or plans for how to lose weight	88 (54)		
	I check the nutritional information of the food I choose when eating out	95 (58)		
Motivation	I use pictures of myself, other people, events, or places to remind me why I want to lose weight	57 (35)	94	45 (24)
	I look at clothes that I would like to wear but are currently too small to boost my motivation to lose weight	90 (55)		
	I keep a note or a record to remind myself of my reasons for losing weight	48 (29)		
	I keep myself motivated by reminding myself of the reasons I want to lose weight	126 (77)		
	I use a chart, graph, or diary to track my progress in losing weight	82 (50)		
	I use an online program or app to look at my body shape and how it could change	21 (13)		
	I draw on my values about what is important in life to motivate me to lose weight	87 (53)		
Planning content	I plan my meals in advance to manage my weight	113 (69)	93	53 (27)
	When I’m away from home (eg, at work), I take food with me so that I can stick to my eating plan	111 (68)		
	If I’m going to eat out, I’ll plan what I’m going to eat before I get there to help me stick to my diet	85 (52)		
	I plan my food shopping in advance to help me stick to my diet (eg, use a shopping list)	110 (67)		
	As part of sticking to my diet, I use particular recipes	105 (64)		
	If I’m trying to get somewhere, I’ll walk or cycle some or all of the way as a way to help me manage my weight (eg, cycling to work, walking to the shops instead of driving or taking the bus)	74 (45)		
	I follow an exercise plan/routine	38 (23)		
	I’ve taken to doing more chores at home/in the garden to help me get more exercise and lose weight	65 (40)		
	I've chosen a type of physical activity I prefer to keep it up	80 (49)		
Scheduling of diet and activity	I plan the times of day when I am going to eat to help me with my weight loss plans	88 (54)	88	37 (25)
	I eat at mealtimes, even if I’m not hungry	70 (43)		
	I go to bed at a particular time each night to help me lose weight	17 (10)		
	I schedule physical activity into my week	79 (48)		
	I give up something else to make time for exercise (eg, watching TV, socializing, leave work early)	54 (33)		
	I try to exercise at times or in places where other people are least likely to see me	40 (24)		
	I plan when I am going to do my food shopping to make sure it's not at a time when I am really hungry	81 (49)		
Regulation: Allowances	My diet allows me to I eat as much as I want of certain types of food and drinks	51 (31)	65	43 (37)
	After I’ve been dieting for a certain amount of time, I’ll allow myself a food treat that isn’t part of my diet	90 (55)		
Regulation: Restrictions	I skip meals as a way to lose weight	60 (37)	88	44 (26)
	To help me manage what I eat, I try to avoid eating certain foods	135 (82)		
	I try not to eat anywhere but at the table	76 (46)		
	Part of my plan to lose weight is to eat only the foods I especially enjoy	52 (32)		
	I avoid eating in certain settings or at certain times of day as a way to help me lose weight (eg, in front of the television, on the go, late at night)	64 (39)		
	There are specific shops or aisles in the supermarket that I avoid to help me stick to my diet	54 (33)		
	To control how much I have, I put a certain amount of food on a plate or drink in a glass	102 (62)		
	I choose not to go out because I am on a diet	57 (35)		
	I think ahead about ways to turn down food when people offer it to me (eg, before going to a party I think of reasons to give for turning down cake)	62 (38)		
	I leave the table immediately after I’ve finished eating as a way to keep me from eating more	43 (26)		
	I avoid eating some of my favorite foods because I’m worried if I start eating I may eat too much	97 (59)		
Regulation: Rule setting	When I go food shopping, I try to only buy the types of food that I know I should be eating	129 (79)	96	55 (25)
	I slow down how quickly I eat in an effort to eat less	94 (57)		
	I drink water or low-calorie/low-sugar drinks between alcoholic drinks to limit the number of calories I consume	91 (55)		
	I cut my food into small pieces to help me manage my eating	46 (28)		
	I have water or a low-calorie drink or eat low-calorie food to help me eat less during meals	111 (68)		
	When I am food shopping, there are certain foods I stay away from to help me stick to my diet	124 (76)		
	I would swap one type of food or drink for another if I knew one would be better for my diet (eg, lower-fat or lower-sugar versions of foods I normally eat)	126 (77)		
	When I’m being active, I push myself to my limits	61 (37)		
	I try to leave food on my plate at the end of a meal as a way to eat less	36 (22)		
Restraint	I have a weight management plan, but I allow myself to be flexible about what I do depending on circumstances	112 (68)	68	68 (47)
Reward	I’ve designated certain times/days as points where I don’t have to stick to my diet, to reward me for sticking to it at other points	35 (21)	62	23 (22)
	I’ll reward myself for my sticking to my exercise goals by occasionally having days where I don’t do much physical activity	33 (20)		
	If I’ve reached one of my weight loss targets, I will reward myself with something that isn't food	73 (45)		
	I put aside money at the beginning of my weight loss attempt and only allow myself to have it back if I’ve hit my targets	9 (5)		
Self-monitoring	I keep track of the calorie or nutritional content of the things I eat	98 (60)	98	54 (23)
	I check the portion sizes of the things I eat	126 (77)		
	I keep track of the physical activity that I do	47 (29)		
	I measure my fitness levels (eg, heart rate, how long it takes to do certain things, how much weight I can lift)	38 (23)		
	I keep track of my weight by weighing myself	130 (79)		
	I measure my waist (or other parts of my body)	59 (36)		
	I use the tightness of my clothes or rings to keep track of my weight	122 (74)		
Stimulus control	If I feel like eating but am trying not to, I avoid going into shops that sell food	76 (46)	88	22 (15)
	I use smaller plates, bowls, or glasses when eating to help with my portion control	98 (60)		
	I buy food pre-packaged in individual portions whenever I can	50 (30)		
	As a way to keep me from overeating, I serve up food before bringing it to the table	84 (51)		
	If I have leftovers, I’ll store them in portion-controlled amounts to prevent me from eating too much later	51 (31)		
	As a way to eat less, I share food when eating out	62 (38)		
	I buy smaller amounts of certain foods to help me eat less	107 (65)		
	To help me manage my weight, I order smaller dishes when eating at a restaurant (eg, a starter or child size dish as a main)	63 (38)		
	To avoid eating or drinking things that don’t fit with my diet, I don’t keep them at home	114 (70)		
	I do something to prompt me to exercise (eg, lay out my exercise clothes the night before)	30 (18)		
	I have systems to remind me to be physically active during the day (eg, reminders on my phone)	23 (14)		
	I buy or prepare healthy foods in a way that makes me more likely to eat them (eg, fruit salad instead of whole fruits, carrot batons instead of whole carrots)	92 (56)		
Support: Buddying	I am trying to lose weight alongside a friend/family member/my partner	44 (27)	28	15 (25)
	I have an online weight loss buddy	5 (3)		
Support: Motivational	I exercise in a group and I think it is more enjoyable than exercising alone	22 (13)	94	38 (20)
	I exercise in a group and it makes me work harder than I would do on my own	33 (20)		
	I’ve tried to get the people I live with to encourage my weight loss plans	117 (71)		
	I’ve tried to get my friends and family to support me in managing my weight	117 (71)		
	I warn people in advance that I am dieting so that they don’t offer me foods I am trying not to eat	74 (45)		
	I told other people about my weight loss goals to help me stick to them	115 (70)		
	I’ve promised other people I'll lose a certain amount of weight	58 (35)		
	I belong to a group of people who are trying to lose weight together (for example, an online discussion forum, a group of colleagues all trying to lose weight)	18 (11)		
	I exercise with a group and when I don’t attend someone asks me why I wasn’t there	9 (5)		
Support: Professional	Feeling stressed, down, or anxious can make me break my diet so I sought help to tackle my feelings	54 (33)	92	56 (29)
	I’ve talked to a health care professional about managing my weight (eg, doctor, nurse, dietitian, physiotherapist, psychologist)	128 (78)		
	I use a weight loss service to help me manage my weight (for example, Weight Watchers™, Slimming World™, Lighter Life™)	92 (56)		
Weight management aids	I use meal replacements (eg, shakes, diet bars, etc.)	95 (58)	72	26 (21)
	I exercise at home using my own equipment or DVDs	39 (24)		
	I use the gym as part of my weight loss efforts	28 (17)		
	I use something at my desk that helps me stay more active when working (eg, a standing desk)	7 (4)		

a Oxford Food and Behaviours (OxFAB) questionnaire.

b Doctor Referral of Overweight People to Low-Energy total diet replacement Treatment (DROPLET).

c Proportion of the participants completing OxFAB (n = 164) who reported use of the strategy.

d Proportion of the participants completing OxFAB (n = 164) who reported using one or more strategies within the domain.

e The mean proportion of strategies used by participants within the domain.

**Supplementary Figure 3:** Associations between individual OxFAB^a^ strategy use and weight change from baseline at 3 months and 1 year in participants in the DROPLET^b^ trial.

Strategy	Weight Change from Baseline to 3 Months		Weight change from baseline to 1 year	
	Mean difference (kg)	95% CI	Mean difference (kg)	95% CI
I use pictures of myself, other people, events, or places to remind me why I want to lose weight	–1.85	(–5.16–1.46)	−0.91	(−4.36–2.54)
I look at clothes that I would like to wear but are currently too small to boost my motivation to lose weight	1.32	(–1.83–4.47)	0.63	(−2.62–3.87)
I keep a note or a record to remind myself of my reasons for losing weight	–2.63	(–6.07–0.82)	−0.28	(−3.88–3.32)
I keep myself motivated by reminding myself of the reasons I want to lose weight	–4.19	(–7.89 to −0.49)	−3.91	(−7.72 to −0.1)
I use a chart, graph, or diary to track my progress in losing weight	–4.30	(–7.39 to −1.21)	−5.54	(−8.73 to −2.34)
I use an online program or app to look at my body shape and how it could change	–1.51	(–6.24–3.23)	−1.69	(−6.99–3.61)
I draw on my values about what is important in life to motivate me to lose weight	−0.30	(−3.43–2.83)	−3.21	(−6.44–0.02)
When I’m grocery shopping and items of food look similar, I make my choice based on the nutritional information on the food labels	−2.20	(−5.58–1.19)	−3.64	(−7.16 to −0.12)
I use a book, website, or app to look up the nutrition information and/or calorie content of the foods I eat	–1.83	(−4.96–1.3)	−1.74	(−4.98–1.5)
I’ve looked up information on how many calories I burn doing physical activity	–1.86	(−5.28–1.55)	−2.48	(−6.02–1.06)
I’ve looked up strategies, tips, or plans for how to lose weight	0.93	(−2.22–4.08)	0.90	(−2.35–4.15)
I exercise with a group and when I don’t attend someone asks me why I wasn’t there	–0.53	(−7.38–6.33)	1.12	(−6.02–8.25)
I exercise in a group, and I think it is more enjoyable than exercising alone	–4.68	(−9.21 to −0.15)	−7.34	(−11.96 to −2.73)
I exercise in a group, and it makes me work harder than I would do on my own	−0.59	(−4.48–3.31)	−0.83	(−4.88–3.23)
I’ve tried to get the people I live with to encourage my weight loss plans	1.92	(−1.53–5.38)	0.13	(−3.42–3.67)
I’ve tried to get my friends and family to support me in managing my weight	0.21	(−3.24–3.67)	−1.67	(−5.21–1.88)
I warn people in advance that I am dieting so that they don’t offer me foods I am trying not to eat	−3.77	(−6.86 to −0.69)	−6.01	(−9.21 to −2.81)
I told other people about my weight loss goals to help me stick to them	0.46	(−2.99–3.91)	0.13	(−3.4–3.65)
I’ve promised other people I'll lose a certain amount of weight	1.13	(−2.16–4.42)	−0.81	(−4.21–2.58)
I belong to a group of people who are trying to lose weight together (for example, an online discussion forum, a group of colleagues all trying to lose weight)	−0.95	(−5.94–4.04)	−3.30	(−8.98–2.38)
I am trying to lose weight alongside a friend/family member/my partner	−0.10	(−3.64–3.45)	0.52	(−3.17–4.21)
I have an online weight loss buddy	1.76	(−7.31–10.84)	−1.41	(−10.49–7.68)
Feeling stressed, down, or anxious can make me break my diet so I sought help to tackle my feelings	2.10	(−1.2–5.41)	5.65	(2.25–9.04)
I’ve talked to a health care professional about managing my weight (eg, doctor, nurse, dietitian, physiotherapist, psychologist)	−0.18	(−3.95–3.59)	0.60	(−3.26–4.46)
I use a weight loss service to help me manage my weight (for example, Weight Watchers™, Slimming World™, Lighter Life™)	−4.02	(−7.08 to −0.96)	−7.31	(−10.46 to −4.15)
I set myself goals for what or how much I’ll eat each day or each week	1.47	(−1.7–4.64)	−0.18	(−3.45–3.09)
I set myself goals for how active I’ll be each day or each week	−1.41	(−4.62–1.8)	−1.60	(−4.9–1.71)
I know how I want to look and won’t give up losing weight until I am the size and shape I want to be	−2.88	(−6.55–0.79)	−4.68	(−8.46 to −0.91)
I have a goal weight in mind that I am working toward	−3.95	(−8.45–0.56)	−9.19	(−13.72 to −4.65)
I set goals that may be unrealistic	−2.71	(−5.84–0.42)	−2.12	(−5.36–1.11)
I want to achieve my weight loss targets within a set amount of time or by a certain date (eg, I want to lose weight in time for my holiday)	−0.11	(−3.3–3.08)	1.45	(−1.84–4.73)
I have a clear goal for the amount of weight I want to lose each week	−1.65	(−4.79–1.49)	−5.07	(−8.3 to −1.84)
I’ve decided to eat some particular foods or do certain types of physical activity in my attempt to lose weight because it worked for someone I know	−0.19	(−3.47–3.1)	2.01	(−1.37–5.39)
I plan the times of day when I am going to eat to help me with my weight loss plans	1.17	(−1.97–4.31)	−1.38	(−4.62–1.87)
I eat at mealtimes, even if I’m not hungry	−0.98	(−4.13–2.16)	−4.45	(−7.7 to −1.2)
I go to bed at a particular time each night to help me lose weight	−2.11	(−7.32–3.1)	−2.88	(−8.31–2.56)
I schedule physical activity into my week	−1.49	(−4.62–1.64)	−2.54	(−5.77–0.7)
I plan my meals in advance to manage my weight	−2.92	(−6.23–0.39)	−6.95	(−10.41 to −3.48)
When I’m away from home (eg, at work), I take food with me so that I can stick to my eating plan	−0.44	(−3.77–2.89)	−2.71	(−6.15–0.73)
If I’m going to eat out, I’ll plan what I’m going to eat before I get there to help me stick to my diet	0.49	(−2.64–3.62)	−0.87	(−4.1–2.37)
I plan my food shopping in advance to help me stick to my diet (eg, use a shopping list)	−2.32	(−5.62–0.98)	−5.20	(−8.6 to −1.79)
As part of sticking to my diet, I use particular recipes	−1.28	(−4.55–1.98)	−0.62	(−3.99–2.75)
If I’m trying to get somewhere, I’ll walk or cycle some or all of the way as a way to help me manage my weight (eg, cycling to work, walking to the shops instead of driving or taking the bus)	0.57	(−2.59–3.72)	−0.53	(−3.79–2.72)
I follow an exercise plan/routine	−2.54	(−6.26–1.18)	−2.90	(−6.71–0.91)
I’ve taken to doing more chores at home/in the garden to help me get more exercise and lose weight	−3.33	(−6.5 to −0.15)	−4.43	(−7.71 to −1.16)
If I feel like eating but am trying not to, I avoid going into shops that sell food	−0.49	(−3.64–2.66)	−0.85	(−4.1–2.41)
I use smaller plates, bowls, or glasses when eating to help with my portion control	−0.01	(−3.21–3.19)	−0.50	(−3.8–2.81)
I buy food pre-packaged in individual portions whenever I can	−0.01	(−3.44–3.41)	1.28	(−2.23–4.8)
As a way to keep me from overeating, I serve up food before bringing it to the table	0.66	(−2.49–3.8)	0.42	(−2.83–3.66)
If I have leftovers, I’ll store them in portion-controlled amounts to prevent me from eating too much later	5.17	(1.86–8.48)	8.26	(4.88–11.64)
As a way to eat less, I share food when eating out	0.43	(−2.81–3.67)	1.51	(−1.82–4.84)
I buy smaller amounts of certain foods to help me eat less	3.55	(0.28–6.82)	4.33	(0.94–7.73)
To help me manage my weight, I order smaller dishes when eating at a restaurant (eg, a starter or child size dish as a main)	2.78	(−0.43–6)	4.14	(0.82–7.46)
To avoid eating or drinking things that don’t fit with my diet, I don’t keep them at home	1.92	(−1.47–5.31)	2.96	(−0.57–6.5)
I do something to prompt me to exercise (eg, lay out my exercise clothes the night before)	1.72	(−2.35–5.79)	0.57	(−3.61–4.75)
I have systems to remind me to be physically active during the day (eg, reminders on my phone)	−2.46	(−6.93–2.02)	−4.32	(−8.93–0.29)
I buy or prepare healthy foods in a way that makes me more likely to eat them (eg, fruit salad instead of whole fruits, carrot batons instead of whole carrots)	1.84	(−1.31–4.99)	3.27	(0.01–6.52)
When I feel like eating something that doesn’t fit with my diet, I acknowledge the feeling but don’t act on it	0.99	(−2.33–4.31)	−1.91	(−5.34–1.52)
When I feel uncomfortable or am in pain when I’m exercising, I accept it as I know it’s doing me good	−2.39	(−5.83–1.04)	−1.69	(−5.22–1.84)
If I feel like eating but am trying not to, I find something else to do to distract myself	−2.34	(−6.06–1.38)	−2.84	(−6.67–0.99)
When I have a craving for something I shouldn’t eat, I try to think about something else to take my mind off it	−3.27	(−6.85–0.31)	−4.94	(−8.67 to −1.21)
If I feel like eating but am trying not to, I make myself wait a certain amount of time to see whether the craving passes	2.85	(−0.39–6.09)	2.68	(−0.66–6.02)
If I feel like eating but am trying not to, I chew gum, drink something, or brush my teeth to stop me from doing so	−0.85	(−4.04–2.34)	0.04	(−3.24–3.32)
I try to go to sleep as a way to not eat when I am feeling hungry	0.53	(−2.89–3.95)	3.38	(−0.18–6.93)
If I feel like I want to stop exercising, I focus on something else to distract me so that I keep going	−0.84	(−4.15–2.48)	−2.79	(−6.2–0.62)
If I feel like eating but am trying not to, I pause and ask myself whether I’m hungry	1.13	(−2.02–4.28)	1.12	(−2.14–4.37)
Partway through eating a meal, I pause and ask myself if I’m full enough to stop	0.51	(−2.66–3.68)	−0.25	(−3.55–3.04)
If I feel like being inactive, I ask myself why I feel that way	−0.01	(−3.67–3.65)	−0.88	(−4.63–2.86)
When I go food shopping, I try to only buy the types of food that I know I should be eating	−1.14	(−4.97–2.68)	−2.45	(−6.4–1.5)
I slow down how quickly I eat in an effort to eat less	−0.23	(−3.4–2.94)	−1.83	(−5.1–1.44)
I drink water or low-calorie/low-sugar drinks between alcoholic drinks to limit the number of calories I consume	3.13	(−0.01–6.27)	3.50	(0.26–6.74)
I cut my food into small pieces to help me manage my eating	2.37	(−1.12–5.86)	−0.31	(−3.95–3.33)
I have water or a low-calorie drink or eat low-calorie food to help me eat less during meals	−0.10	(−3.44–3.23)	−2.46	(−5.89–0.98)
When I am food shopping, there are certain foods I stay away from to help me stick to my diet	−1.13	(−4.77–2.5)	−0.04	(−3.83–3.74)
I would swap one type of food or drink for another if I knew one would be better for my diet (eg, lower fat or lower sugar versions of foods I normally eat)	0.17	(−3.53–3.87)	−1.82	(−5.68–2.05)
When I’m being active, I push myself to my limits	−1.87	(−5.11–1.37)	−2.16	(−5.48–1.17)
I try to balance how much I eat, so if eat a lot at one point I’ll make up for it by eating less at other points	0.92	(−2.65–4.49)	1.71	(−1.96–5.39)
I adjust how much I eat depending on how much physical activity I’ve done or plan to do	−0.36	(−3.53–2.81)	1.20	(−2.08–4.47)
I adjust how much physical activity I do depending on how much I’ve eaten or am planning to eat later	−0.08	(−4.07–3.91)	1.50	(−2.71–5.7)
If I haven’t been very active, or know I’m going to be inactive for a while, I’ll exercise more at another point–balance it out	−2.87	(−6.42–0.68)	−6.30	(−9.95 to −2.65)
I skip meals as a way to lose weight	−0.15	(−3.41–3.1)	1.42	(−1.94–4.79)
To help me manage what I eat, I try to avoid eating certain foods	3.77	(−0.31–7.85)	3.66	(−0.57–7.89)
I try not–eat anywhere but at the table	−0.11	(−3.26–3.04)	−0.73	(−3.98–2.52)
Part of my plan to lose weight is to eat only the foods I especially enjoy	1.54	(−1.84–4.92)	1.94	(−1.54–5.42)
I avoid eating in certain settings or at certain times of day as a way to help me lose weight (eg, in front of the television, on the go, late at night)	0.27	(−2.95–3.49)	−0.57	(−3.89–2.76)
There are specific shops or aisles in the supermarket that I avoid to help me stick to my diet	0.45	(−2.89–3.8)	1.12	(−2.34–4.58)
To control how much I have, I put a certain amount of food on a plate or drink in a glass	1.62	(−1.61–4.84)	−0.20	(−3.57–3.17)
I choose not to go out because I am on a diet	−1.33	(−4.63–1.97)	−1.43	(−4.83–1.97)
I think ahead about ways to turn down food when people offer it to me (eg, before going to a party I think of reasons to give for turning down cake)	1.77	(−1.45–4.99)	−1.01	(−4.36–2.34)
I leave the table immediately after I’ve finished eating as a way to keep me from eating more	−0.12	(−3.69–3.45)	−1.54	(−5.28–2.2)
My diet allows me to eat as much as I want of certain types of food and drinks	1.69	(−1.69–5.06)	3.10	(−0.4–6.61)
I keep track of the calorie and/or nutritional content of the things I eat	−2.00	(−5.19–1.19)	−2.24	(−5.55–1.07)
I check the portion sizes of the things I eat	1.80	(−1.92–5.53)	1.23	(−2.7–5.15)
I keep track of the physical activity that I do	−3.07	(−6.52–0.37)	−4.45	(−8.01–-0.9)
I measure my fitness levels (eg, heart rate, how long it takes to do certain things, how much weight I can lift)	0.10	(−3.63–3.83)	0.59	(−3.22–4.4)
I keep track of my weight by weighing myself	−1.90	(−5.74–1.94)	−4.31	(−8.24–-0.38)
I measure my waist (or other parts of my body)	−2.07	(−5.32–1.19)	−2.56	(−5.93–0.82)
I use the tightness of my clothes or rings to keep track of my weight	−2.93	(−6.5–0.63)	−3.89	(−7.56 to −0.22)
I have a weight management plan, but I allow myself to be flexible about what I do depending on circumstances	−1.73	(−5.09–1.62)	−0.09	(−3.57–3.39)
After I’ve been dieting for a certain amount of time, I’ll allow myself a food treat that isn’t part of my diet	0.70	(−2.44–3.84)	2.05	(−1.19–5.29)
I’ve designated certain times/days as points where I don’t have to stick to my diet, to reward me for sticking to it at other points	−1.17	(−4.99–2.64)	0.37	(−3.66–4.4)
I’ll reward myself for my sticking–my exercise goals by occasionally having days where I don’t do much physical activity	−1.40	(−5.29–2.48)	0.31	(−3.77–4.4)
If I’ve reached one of my weight loss targets, I will reward myself with something that isn't food	−0.35	(−3.52–2.81)	−0.30	(−3.59–2.98)
I put aside money at the beginning of my weight loss attempt and only allow myself to have it back if I’ve hit my targets	−1.79	(−8.64–5.07)	1.40	(−5.74–8.54)
I use meal replacements (eg, shakes, diet bars, etc.)	−2.47	(−5.57–0.63)	−4.91	(−8.12 to −1.71)
I exercise at home using my own equipment or DVDs	−0.13	(−3.83–3.56)	−1.46	(−5.25–2.32)
I use the gym as part of my weight loss efforts	0.77	(−3.4–4.95)	3.59	(−0.66–7.84)
I use something at my desk that helps me stay more active when working (eg, a standing desk)	3.20	(−4.89–11.29)	−3.15	(−11.25–4.95)
I've chosen a type of physical activity I prefer to keep it up	0.70	(−2.43–3.84)	−0.83	(−4.07–2.41)
I give up something else to make time for exercise (eg, watching TV, socializing, leave work early)	−4.14	(−7.43––0.86)	−6.91	(−10.27 to −3.55)
I try to exercise at times or in places where other people are least likely to see me	1.51	(−2.15–5.17)	1.19	(−2.58–4.96)
I check the nutritional information of the food I choose when eating out	0.68	(−2.5–3.86)	1.00	(−2.28–4.29)
I plan when I am going to do my food shopping to make sure it's not at a time when I am really hungry	0.59	(−2.55–3.73)	0.90	(−2.34–4.15)
I try to leave food on my plate at the end of a meal as a way to eat less	1.34	(−2.46–5.14)	2.53	(−1.35–6.42)
I avoid eating some of my favorite foods because I’m worried if I start eating I may eat too much	0.51	(−2.68–3.7)	0.48	(−2.81–3.77)

a Oxford Food and Behaviours questionnaire (OxFAB) assesses 117 (only 115 used in this study) cognitive and behavioural strategies, grouped into 21 domains used by individuals losing weight.

b Doctor Referral of Overweight People to Low-Energy total diet replacement Treatment (DROPLET).

**Supplementary Figure 4:** Data-driven patterns of strategies of the OxFAB^a^ questionnaire used by participants from the DROPLET^b^ trial.

	Description	Included strategies	Factor loading score
#1	Physical Activity	I schedule physical activity into my week	0.2596
		I've chosen a type of physical activity I prefer to keep it up	0.2451
		I set myself goals for how active I’ll be each day or each week	0.2385
		If I’m trying to get somewhere, I’ll walk or cycle some or all of the way as a way to help me manage my weight (eg, cycling to work, walking to the shops instead of driving or taking the bus)	0.2360
		When I’m being active, I push myself to my limits	0.2278
		If I feel like I want to stop exercising, I focus on something else to distract me so that I keep going	0.2256
		When I feel uncomfortable or am in pain when I’m exercising, I accept it because I know it’s doing me good	0.2247
		I give up something else to make time for exercise (eg, watching TV, socializing, leave work early)	0.2113
		I keep track of the physical activity that I do	0.2089
		I follow an exercise plan/routine	0.2026
#2	Motivation	I know how I want to look and won’t give up losing weight until I am the size and shape I want to be	0.2912
		I have an online weight loss buddy	0.2605
		I put aside money at the beginning of my weight loss attempt and only allow myself to have it back if I’ve hit my targets	0.2341
		I go to bed at a particular time each night to help me lose weight	0.2173
		I want to achieve my weight loss targets within a set amount of time or by a certain date (eg, I want to lose weight in time for my holiday)	0.2133
		I keep myself motivated by reminding myself of the reasons I want to lose weight	0.2132
#3	Planned Eating	I use meal replacements (eg, shakes, diet bars, etc.)	0.3202
		I use a weight loss service to help me manage my weight (for example, Weight Watchers, Slimming World, Lighter Life)	0.2999
		I choose not to go out because I am on a diet	0.2108
		When I’m away from home (eg, at work), I take food with me so that I can stick to my eating plan	0.2089
#4	Food Purchasing	When I am food shopping, there are certain foods I stay away from to help me stick to my diet	0.3764
		When I go food shopping, I try to only buy the types of food that I know I should be eating	0.3594
		When I’m grocery shopping and items of food look similar, I make my choice based on the nutritional information on the food labels	0.337
		I plan my food shopping in advance to help me stick to my diet (eg, use a shopping list)	0.3016
		I plan when I am going to do my food shopping to make sure it's not at a time when I am really hungry	0.3016
		I exercise with a group and when I don’t attend someone asks me why I wasn’t there	0.2071
#5	N/A		None above 0.2

a Oxford Food and Behaviours (OxFAB) questionnaire.

b Doctor Referral of Overweight People to Low-Energy total diet replacement Treatment (DROPLET).
